# NLOS Identification and Error Compensation Method for UWB in Workshop Scene

**DOI:** 10.3390/s25216555

**Published:** 2025-10-24

**Authors:** Yu Su, Quan Yu, Xiaohao Xia, Wenfeng Li, Lijun He, Taiwei Yang

**Affiliations:** 1 Zhoushan COSCO Shipping Heavy Industry Co., Zhoushan 316100, China; 2 School of Transportation and Logistics Engineering, Wuhan University of Technology, Wuhan 430063, China; liwf@whut.edu.cn

**Keywords:** UWB, NLOS identification, error compensation, maximum likelihood estimation, adaptive extended Kalman filter

## Abstract

To address the frequent safety incidents caused by positioning uncertainty due to NLOS (Non-Line-of-Sight) interference in complex manufacturing workshop environments, this paper aims to achieve high-precision distance measurement and positioning in complex workshop scenarios. First, common NLOS identification methods are analyzed. By combining received signal energy and ranging residuals, a rapid NLOS identification method is proposed. Building on this foundation, a ranging error compensation method based on maximum likelihood estimation and adaptive extended Kalman filtering is designed. Finally, static experiments are conducted to verify the effectiveness of the proposed NLOS identification method and ranging error compensation approach. Experimental results indicate that the ranging accuracy of the proposed method has been significantly improved and demonstrates considerable advantages over traditional Kalman filtering algorithms.

## 1. Introduction

**Figure 1:**
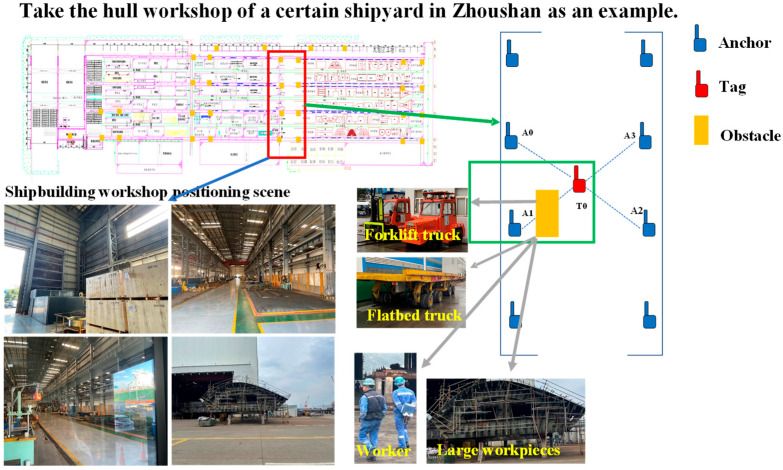
Schematic representation of workshop localization scenarios and associated interference factors.

Ultra-Wideband (UWB) technology is a versatile radio frequency (RF) technology [1,2] that uses nanosecond-level non-sinusoidal narrow pulses for data transmission. It features high multipath resolution, strong penetration capability, and high positioning accuracy [3,4], and is widely applied in positioning, ranging, and target detection in indoor and complex environments [5]. The key challenge in UWB positioning technology lies in accurately estimating the actual distance between a tag and an anchor node using information such as received signal strength, time-of-flight, and signal arrival angle from UWB anchor nodes [6]. Generally, UWB ranging accuracy is affected by internal and external factors. Internal factors include hardware interference within the system and errors in built-in algorithms; external factors primarily involve non-line-of-sight (NLOS) environmental interference [7]. Internal errors can be effectively corrected through algorithm improvements, but external errors are difficult to eliminate in practical scenarios [8,9]. For example, in a large shipyard manufacturing enterprise, metal components, moving personnel, and vehicles within the processing workshop can cause UWB anchors to be in NLOS states, significantly interfering with ranging accuracy. Due to location uncertainty, safety accidents frequently occur. Therefore, research on indoor high-precision ranging and positioning holds great practical significance. The workshop application scenario is illustrated in Figure 1. In the four-anchor-one-tag positioning system (A0, A1, A2, A3, T0), distance measurements in the A1-T0 section exhibit a positive bias. The extent of this bias depends on the material, size, and reflective properties of NLOS obstructions. Additionally, due to unpredictable personnel, workpiece, and vehicle movements, real-time positioning systems relying solely on algorithms struggle to maintain high stability and risk positioning failure.

Regarding NLOS recognition and ranging error compensation, numerous solutions have been proposed and applied. Liu et al. [10] proposed a Hard Sample Meta Learning (HSML) framework to address the cross-scene and cross-domain challenges of UWB NLOS recognition. The method consists of two stages: the meta-training stage employs dual-cycle learning and residual hard sample mining, combined with residual correction focal loss to enhance robustness; the meta-testing stage avoids the influence of abnormal samples through a residual trend filtering mechanism. Experiments show that HSML significantly outperforms existing methods on multiple real-world datasets, achieving high accuracy, strong robustness, and rapid adaptation. Deng [11] proposed a novel NLOS identification and mitigation method based on a multi-input parallel deep learning model and Gramian angular field (GAF). In the model training phase, CNN was used to extract temporal features from the original CIR signal, and the residual network was used to extract visual features from the GAF-encoded image. The proposed method is innovative, but it also increases the complexity, and there is room for further improvement. Wang et al. [12] addressed the challenges of insufficient generalization capability in dynamic environments, modeling complexity, and limited accuracy in UWB-based NLOS identification. They proposed an identification method based on the fusion of a Markov Transition Field and a CNN. Experimental results demonstrate that the proposed method significantly outperforms conventional CNN, SVM, and LSTM methods in NLOS identification. Zhou et al. [13] introduced a joint NLOS identification method using the position difference between the first path and strongest path, the power difference between the received signal and first path, and distance residuals. This achieved 94.52% average identification accuracy in NLOS scenarios.

For ranging error compensation, Chiasson et al. [14] addressed high hardware costs, significant errors, and privacy leaks from clock synchronization in traditional UWB hyperbolic positioning. They proposed an asynchronous hyperbolic UWB source localization method using a message mutual observation mechanism between anchor nodes and a novel TDOA equation reconstruction strategy. This utilized time differences between anchor node transmission/reception to eliminate clock offset via closed-loop equations, with a derived clock drift error boundary model for error correction. Xu et al. [15] tackled accuracy degradation from noise uncertainty and linearization rounding errors in UWB robot localization, proposing a distributed adaptive iterative extended unbiased finite impulse response filtering algorithm based on expectation maximization. Building upon UWB positioning, Juston et al. [16] utilized an improved Sage-Husa Fuzzy Adaptive Filter and a robust Error-State Kalman Filter to process integrated motion inputs from IMU and wheel encoders. This approach effectively mitigates positioning drifting errors and outlier interference in dynamic environments, significantly enhancing the accuracy and robustness of the localization system. Wang et al. [17] proposed an NLOS identification and compensation scheme for dynamic targets using inverse estimation of known anchors and an improved robust unscented Kalman filter, fusing gyroscope/accelerometer data to reduce localization errors.

While these methods achieve effective LOS/NLOS identification and NLOS error compensation, they exhibit high computational complexity and require prior environmental information, limiting robustness across environments. To address this, we design a computationally efficient NLOS identification method that rapidly distinguishes NLOS conditions using UWB ranging distance residuals and energy attenuation changes. We further propose an efficient ranging error compensation method based on probability estimation and filtering.

The main contributions of this paper are as follows:

1. Addressing the high computational complexity of existing methods, this paper designs a computationally efficient recognition approach. By integrating two key features—distance residual and signal energy attenuation—it achieves rapid recognition in NLOS scenarios.
2. Addressing the limitations of existing methods that rely on prior environmental knowledge and exhibit poor cross-environment adaptability, the proposed approach requires no pre-acquired environmental information. Its adaptive algorithm mechanism ensures robust recognition performance in unknown or dynamically changing scenarios, significantly enhancing cross-scenario applicability.
3. For error compensation, this paper proposes an efficient algorithm based on probabilistic estimation and filtering techniques. This mechanism models the statistical characteristics of errors using probabilistic models and combines filtering algorithms to achieve precise suppression of NLOS errors.

The paper is structured as follows: Section 1 outlines UWB communication ranging principles. Section 2 details parameter selection and principles for UWB NLOS identification. Section 3 introduces the ranging error compensation method using Maximum Likelihood Estimation (MLE) and an improved extended Kalman filter. Section 4 validates the method through experimental tests. Section 5 concludes the study.

## 2. UWB Ranging Principle

**Figure 2:**
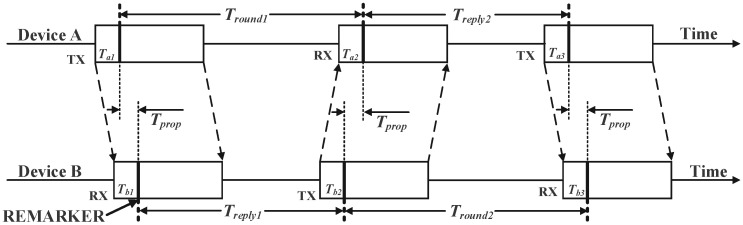
DS-TWR Distance measurement principle diagram.

UWB technology uses non-sinusoidal narrow pulses for data transmission, offering advantages such as high multipath resolution, strong penetration capability, and high ranging accuracy [18]. A common UWB ranging method is the double-sided two-way ranging (DS-TWR) technique, which calculates distances precisely through four signal transmissions [19]. This method mitigates issues like time asynchrony between UWB anchors and reduces clock drift. The DS-TWR ranging principle is illustrated in Figure 2.

First, Device A transmits a signal to Device B. After a propagation delay of *T_prop_*, Device B receives the signal and transmits a response to Device A after a fixed delay *T_reply_*_1_. Upon receiving this response, Device A transmits a final signal to Device B after a fixed delay *T_reply_*_2_. Device B receives this signal, completing the exchange. The theoretical time-of-flight measurement between Device A and Device B is given by:

(1) $$T_{prop}=\frac{T_{round1}\times T_{round2}-T_{reply1}\times T_{reply2}}{T_{round1}+T_{round2}+T_{reply1}+T_{reply2}}$$

Among these, *T_round_*_1_ is the time interval from Device A transmitting a signal to Device B until receiving Device B’s response, and *T_round_*_2_ is the time interval from Device B receiving Device A’s initial signal until receiving Device A’s subsequent signal. *T_reply_*_1_ is the time interval between Device B receiving a signal and transmitting its response, and *T_reply_*_2_ is the time interval between Device A receiving Device B’s response and transmitting its next signal.

The actual distance *d* between Device *A* and Device *B* is calculated as:

(2) $$D_{AB}=d\times (T_{prop}+t_{e})$$

In this equation, *c* represents the speed of light, and *t_e_* represents the systematic error. External interference may cause the measured *T_prop_* value to exceed the true propagation delay, and the correction methods for this will be discussed in detail in subsequent sections.

## 3. NLOS Recognition Method Based on Distance Residual and Energy Difference

Communication between UWB anchors is facilitated by the DW1000 module. Successful communication requires the received signal strength to exceed the receiver’s sensitivity threshold. When this condition is met, the receiver can detect the incoming signal, enabling communication. The received signal strength depends on multiple factors, as quantified by the Friis transmission equation [18]:

(3) $$P_{R}[dBm]=P_{T}[dBm]+G[dB]-L[dB]-2\log_{10}(4\pi f_{c}d/c)-P_{NLOS}[dBm]$$

In this formula, *P_R_* represents the received signal power, *P_T_* denotes the transmitted power, and *G* incorporates the antenna gains of the transmitter and receiver, along with additional gain from external amplifiers. In uncalibrated systems, UWB is constrained by a −41.3 dBm/MHz EIRP PSD limit. *L* represents system losses from PCBs, wires, connectors, etc., while *c* is the speed of light (299,792,458 m/s). *f_c_* is the channel center frequency, *d* is the distance between transmitter and receiver, and *P_NLOS_* denotes the signal attenuation due to NLOS obstructions. Under Line-of-Sight (LOS) conditions, *P_NLOS_* = 0.

When NLOS obstructions occur within the Fresnel zone between anchors, the resulting signal attenuation reduces the received signal strength. This may cause the signal to fall below the receiver’s sensitivity threshold, preventing communication. The magnitude of NLOS attenuation far exceeds signal variations from distance changes. Within workshops, primary NLOS sources include large metal workpieces, human obstructions, and operational vehicles. These obstructions cause significant signal degradation, enabling high NLOS detection sensitivity. By analyzing low-level register parameters from anchor nodes, received signal strength values allow rapid and direct detection of NLOS obstructions.

**Figure 3:**
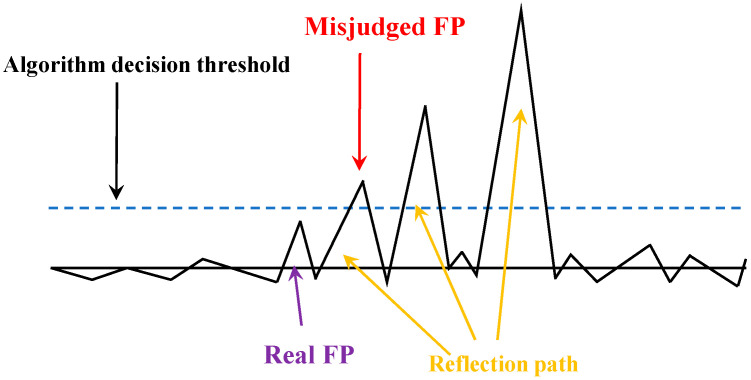
Illustration of sources of error.

In LOS scenarios, the strongest path of the UWB signal is closely aligned with the first path in the accumulator window. While in NLOS scenarios, the strongest path typically appears on the right side of the accumulator window, exhibiting significant positional deviation from the first path, as shown in Figure 3. Since UWB ranging employs a leading-edge detection algorithm, the target position is determined by the first path’s accumulator position that exceeds the system-set threshold. Consequently, when the communication environment transitions between LOS and NLOS states, the attenuated first path may fall below the detection threshold. This causes the system to misidentify the strongest path as the first path, generating substantial distance residuals. These residuals represent the difference between the current distance measurement or optimal distance estimate and the corresponding value from the previous moment:

(4) $$\delta \left(k\right)=d\left(k\right)-d\left(k|k-1\right)$$

where *δ*(*k*) represents the distance residual at time *k*, *d(k)* denotes the distance measurement at time *k* or the current time, and *d*(*k*|*k−*1) indicates the optimal distance estimate at time *k*−1 or the previous time. Using the residual *δ*(*k*) from Equation (4), we formulate the following LOS/NLOS binary hypothesis test:

(5) $$\left\{\begin{array}{ccc} H_{0}: & \delta <\zeta \sigma_{s}^{2} & \mathrm{LOS}\\ H_{1}: & \delta \geq \zeta \sigma_{s}^{2} & \mathrm{NLOS} \end{array}\right.$$

Here, ζ denotes the weight value, a scaling factor used to integrate the binary results of the energy test and the residual test [20]. The threshold *s* is empirically determined based on the residual variance observed during the known LOS calibration phase in the initial stage of the experiments. Specifically, *s* is set as a multiple of the variance of *δ*(*k*) calculated in this initial LOS segment.

This paper proposes an identification method based on the fusion of instantaneous energy attenuation and distance residual features for LOS/NLOS state discrimination of UWB signals in complex workshop environments. These two features capture channel variations from different perspectives—signal strength and ranging consistency—and thus possess inherent complementarity. By simultaneously monitoring sudden energy drops and abnormal residual fluctuations, and employing a threshold-based decision mechanism, the system can effectively overcome the limitations of single-feature approaches in specific scenarios, thereby significantly enhancing the robustness and environmental adaptability of the identification algorithm.

## 4. Ranging Error Compensation Method Based on NLOS Recognition

To address transient NLOS interference in workshop scenarios, this section proposes a UWB ranging error compensation method combining MLE and Adaptive Extended Kalman Filter (AEKF), building upon the NLOS detection framework.

The Kalman Filter (KF) applies to linear dynamic systems. Under partial system state observability, it recursively estimates system states using dynamic models and measurement data, enabling real-time state tracking. Since UWB positioning systems exhibit nonlinear state evolution and observations, an Extended Kalman Filter (EKF) is required. The system state and observation equations are described by nonlinear functions:

(6) $$x_{k}=f(x_{k-1},u_{k-1})+w_{k-1}$$

(7) $$z_{k}=h(x_{k})+v_{k}$$

In this model, *f*() is a nonlinear function describing state evolution over time, while *h*() is a nonlinear function mapping states to observations. EKF operates through two sequential stages: prediction and update.

**Prediction Step:**
(8) $${\overset{⌢}{x}}_{k|k-1}=f({\overset{⌢}{x}}_{k-1|k-1},u_{k-1})$$
(9) $$P_{k|k-1}=F_{k-1}P_{k-1|k-1}F_{k-1}^{T}+Q_{k-1}$$

**Update Step:**
(10) $$K_{k}=P_{k|k-1}H_{k}^{T}{\left(H_{k}P_{k|k-1}H_{k-1}^{T}+R_{k}\right)}^{-1}$$
(11) $${\overset{⌢}{x}}_{k|k}={\overset{⌢}{x}}_{k|k-1}+K_{k}(z_{k}-h({\overset{⌢}{x}}_{k|k-1}))$$
(12) $$P_{k|k}=(I-K_{k}H_{k})P_{k|k-1}$$

In this state-space model, *x_k_* is the state vector at time *k*, *F*_*k*−1_ is the state transition matrix governing state evolution from *k* − 1 to *k*, *B*_*k*−1_ is the control input matrix mapping control input *u_k_*_−1_ to state change, *w_k_*_−1_ is the process noise, assumed zero-mean Gaussian with covariance *Q_k_*_−1_, *z_k_* is the measurement vector, *H_k_* is the observation matrix relating state to measurements, *v_k_* is the measurement noise, assumed zero-mean Gaussian with covariance *R_k_*.

Accounting for large-scale fading, the energy-based ranging model at time *k* is expressed as:

(13) $$P_{R}(k)=10\eta \mathrm{lg}(\frac{d(k)}{d_{0}})+Q$$

In this equation, *η* denotes the path loss exponent, *d*_0_ is the reference distance (typically 1 m), *d*(*k*) represents the measured distance, and the shadowing attenuation *Q* follows a Gaussian distribution with zero mean and variance *λ*^2^. Both the path loss exponent and variance depend on the measurement environment. Parameter values recommended by the IEEE 802.15.4a working group are provided in Table 1 [20]. Dependent on distance measurements, the likelihood function for the energy-ranging model observations is:

(14) $$L(P_{R}(k)|d(k))=\frac{1}{\sqrt{2\pi }\lambda^{2}}e^{\left(-\frac{{\left(P_{R}(k)-10\eta \mathrm{lg}(d(k))\right)}^{2}}{2\lambda^{2}}\right)}$$

By deriving this function, we obtain the MLE of *d*(*k*). This MLE is computed using LOS/NLOS identification results to determine the specific distance value, which serves as the distance input for the AEKF.

(15) $$d(k)=\left\{\begin{array}{ll} 10^{\frac{P_{R}(k)}{10\eta_{LOS}}} & \mathrm{LOS}\\ 10^{\frac{P_{R}(k)}{10\eta_{NLOS}}} & \mathrm{NLOS} \end{array}\right.$$

The ranging measurement distance residual is expressed as:

(16) $$\xi =d(k)-h(x_{k|k-1})$$

The distance residual covariance matrix and recursive covariance matrix are defined as follows:

(17) $$P_{k}^{Res}=E[\xi \xi^{T}]$$

(18) $$P_{k}^{c}=H_{k}P_{k|k-1}H_{k}^{T}+R_{k}$$

To ensure the filtering algorithm compensates exclusively for NLOS data, the discriminant factor *j_k_* is defined as:

(19) jk=trace(PkRes)trace(Pkc)

Using *j_k_*, the observation noise is dynamically adjusted:

(20) $${\tilde{R}}_{k}=\left\{\begin{array}{ll} 1 & j_{k}<e_{0}\\ j_{k}^{m}\times {\left[\frac{e_{1}-e_{0}}{j_{k}-e_{0}}\right]}^{n} & e_{0}\leq j_{k}\leq e_{1}\\ +\infty & j_{k}>e_{1} \end{array}\right.$$

where *e*_0_ and *e*_1_ are threshold parameters. The updated Kalman gain becomes:

(21) $${\tilde{K}}_{k}=P_{k|k-1}H_{k}^{T}{\left(H_{k}P_{k|k-1}H_{k-1}^{T}+{\tilde{R}}_{k}\right)}^{-1}$$

**Figure 4:**
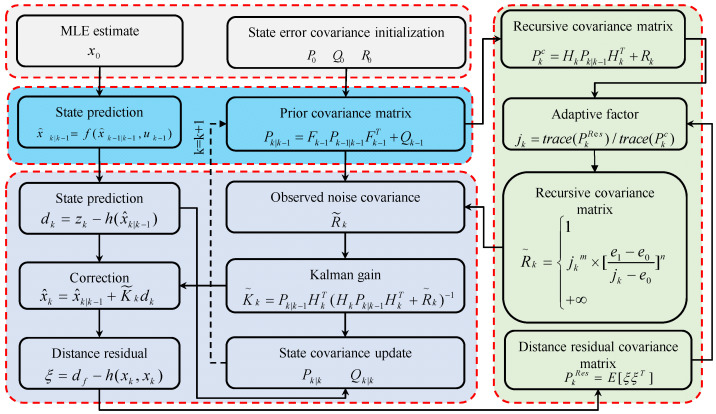
AEKF algorithm workflow.

The adjusted observation noise feeds back into the Kalman gain for NLOS distance compensation, as shown in Figure 4. MLE-processed distance values initialize the AEKF. By analyzing the ratio between the distance residual covariance and recursive covariance matrices for current measurements and prior predictions, the adaptive factor is derived. Based on magnitude, the observation noise is dynamically scaled to update the Kalman gain, correcting distance measurements.

## 5. Experimental Verification

### 5.1. Experimental Setup

**Figure 5:**
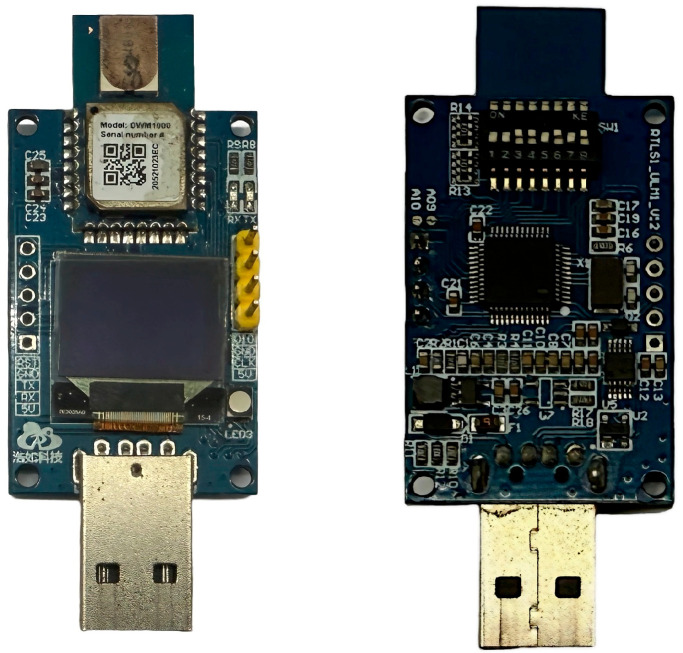
Experimental apparatus.

This section selects appropriate scenarios to conduct UWB experiments validating NLOS/LOS transitions and error compensation methods. Experimental equipment utilizes DecaWave’s DW1000 module with an STM32F103 main controller. In LOS conditions, specifications include ranging accuracy of ±5 cm, positioning frequency of 15 ms, transmission power of −39 dBm/MHz, transmission gain of 20 dB, bandwidth of 500 MHz, and communication rate of 110 K/6.8 MHz. The experimental apparatus is shown in Figure 5.

**Figure 6:**
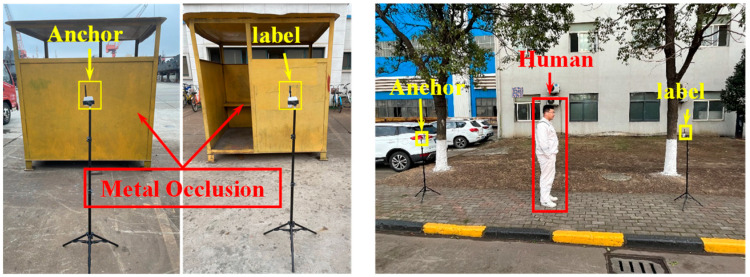
Experimental scenario.

To validate the NLOS identification method’s effectiveness and robustness, experiments were conducted at 2.6–3 m ranges using two obstruction categories: human and metal. Each NLOS obstruction event was brief and repeated multiple times, representing common workshop interference scenarios. Parameter settings, calibrated using the sample variance of the static segment of LOS, configured the ranging system as a single-mode system with state transition matrix = 1, process noise covariance is 0.015 m^2^, and measurement noise covariance is 0.542 m^2^. Figure 6 presents some physical photographs that illustrate the experimental process.

### 5.2. Experimental Analysis

**Figure 7:**
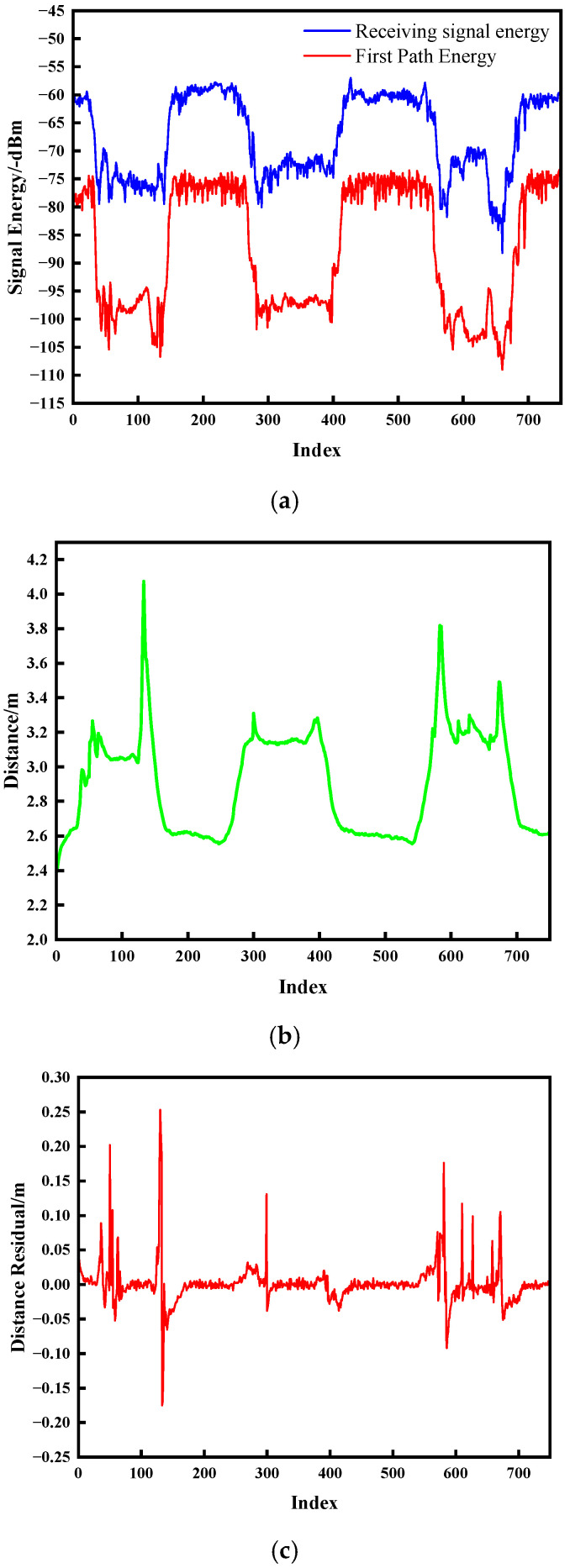
Experimental results for human obstruction. (**a**) Signal energy; (**b**) Change in distance; (**c**) Distance residual.

**Figure 8:**
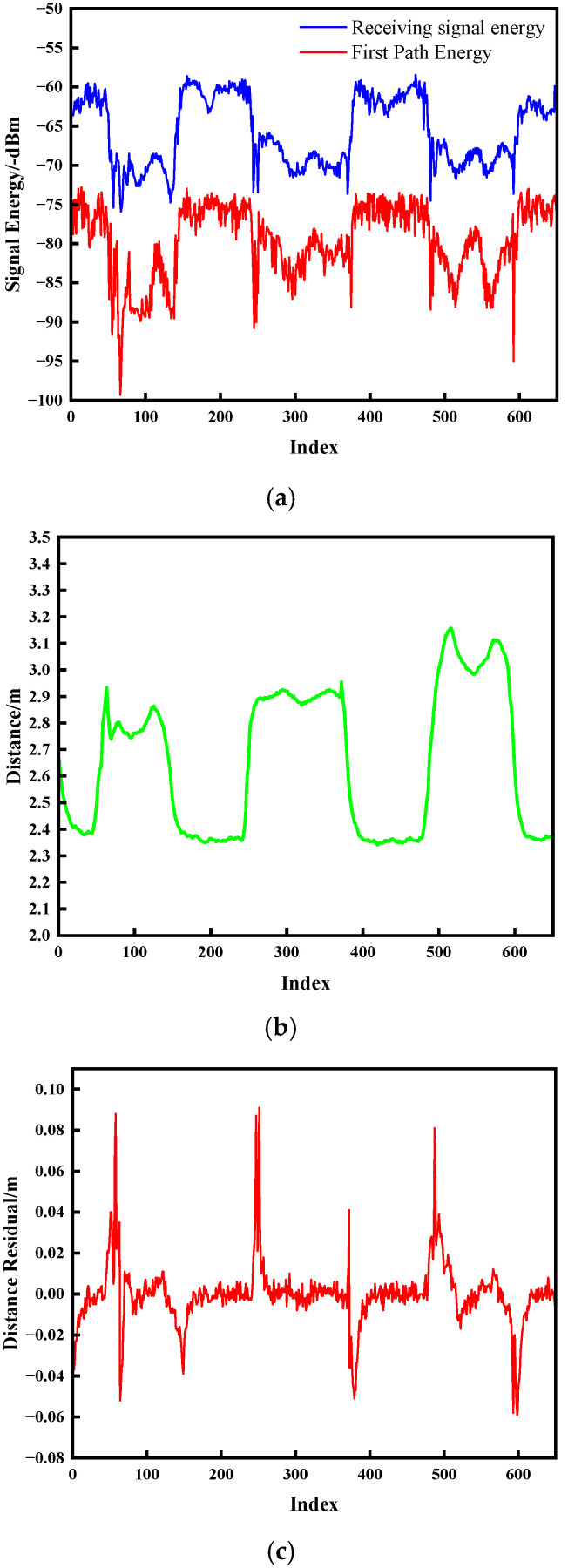
Metallic obstruction test outcomes. (**a**) Signal energy; (**b**) Change in distance; (**c**) Distance residual.

Figure 7 and Figure 8 present the experimental outcomes for dynamic NLOS detection. Subfigure ensembles (a) illustrate temporal energy variations, (b) depict ranging distance trajectories, and (c) document distance residual fluctuations.

As evidenced in Figure 7 and Figure 8, the onset of NLOS obstruction induces severe signal energy attenuation, manifesting as transient convex protrusions that deviate markedly from nominal trajectory baselines. The temporal persistence of these protrusions exhibits direct correlation with obstruction duration. Upon obstruction clearance, energy profiles converge to pre-interference trajectories. Crucially, distance residuals demonstrate pronounced discontinuities during both ingress and egress of NLOS conditions, with peak amplitudes temporally synchronized with energy anomalies and ranging aberrations. This characteristic correlation empirically validates the methodology’s efficacy in detecting environmental transitions between UWB communication nodes.

**Figure 9:**
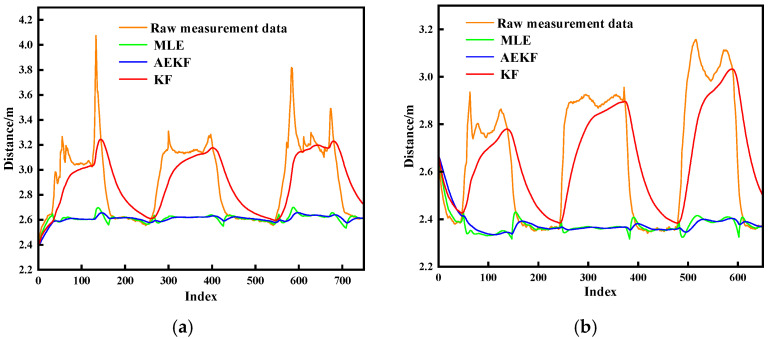
Error compensation experimental results. (**a**) Human body obstruction; (**b**) Metallic obstruction.

Leveraging NLOS detection, Figure 9 presents compensated ranging performance using the proposed MLE-AEKF framework. Figure 9a,b quantify compensation efficacy for anthropomorphic and metallic obstructions, respectively. The ranging values obtained after MLE fitting significantly mitigate interference-induced ranging errors, driving the distance curve to asymptotically approach static LOS baseline measurements. However, the compensated data still exhibits sub-centimeter residual peak deviations. The AEKF-processed output effectively suppresses these artifacts through dynamic Kalman gain modulation via adaptive scaling factors, substantially reducing peak-induced interference. The KF algorithm (red lines in the figures), widely employed for error mitigation, fails to correct ranging errors under sustained NLOS interference conditions. In contrast, the proposed algorithm delivers superior compensation performance in obstructed environments. Compensated distance values achieve near-LOS accuracy, with specific error reduction rates of 93.75% for anthropomorphic obstructions and 93.11% for metallic obstructions compared to traditional KF implementations.

**Figure 10:**
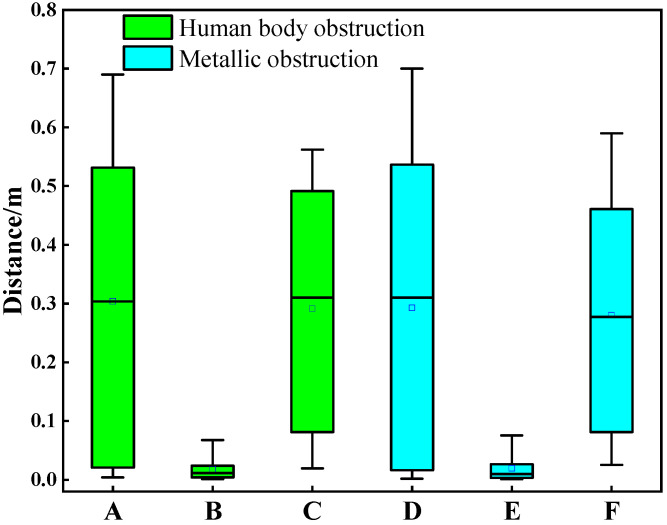
Ranging error distributions across multiple NLOS sources.

In NLOS scenarios involving both anthropomorphic and metallic line-of-sight obstructions, Figure 10 presents error distribution plots for MLE, AEKF, and traditional KF algorithms. Here, A denotes original ranging data under anthropomorphic obstruction, B represents processed distance measurements from our proposed algorithm under identical anthropomorphic conditions, and C indicates measurements processed by conventional KF filtering under anthropomorphic obstruction. Similarly, D signifies original ranging data under metallic obstruction, E corresponds to processed measurements from our algorithm under metallic obstruction, and F designates measurements processed by traditional KF under metallic obstruction. The proposed algorithm achieves ranging accuracies of 1.82 cm and 1.93 cm under anthropomorphic and metallic obstructions, respectively, with maximum absolute ranging errors of 9.24 cm and 9.83 cm, respectively. Compared to unprocessed ranging data, our method improves ranging accuracy by 93.91% and 93.41% under anthropomorphic and metallic obstructions, respectively. Relative to traditional KF filtering, the proposed algorithm demonstrates accuracy improvements of 93.75% and 93.11% under anthropomorphic and metallic obstructions, respectively.

## 6. Conclusions

To mitigate positional uncertainty in complex industrial machining environments, this paper employs UWB energy parameters and distance residuals as discriminative features for NLOS identification. Building upon this framework, we propose a ranging error compensation method integrating MLE with an AEKF. By dynamically weighting NLOS measurements to reduce their confidence contribution, the algorithm minimizes NLOS-induced error propagation, significantly enhancing ranging stability and accuracy. Experimental validation through static NLOS identification and obstruction-specific compensation tests (anthropomorphic and metallic) confirms the method’s efficacy under realistic workshop conditions.

Although the proposed method proved effective in the presented static experiments, its performance in fully dynamic environments warrants further investigation. Future work will include (1) testing with moving tags and dynamic obstructions to evaluate tracking performance; (2) extending the validation to longer ranges and different areas of a workshop; and (3) incorporating additional types of NLOS obstructions (e.g., concrete, plastic) to further validate the generalizability of the identification and compensation algorithm.

## Figures and Tables

**Table 1:** Parameter values.

Parameters	Enclosed Built Environments		Open-Air Environments	
	LOS	NLOS	LOS	NLOS
*λ*	1.79	2.70	1.76	2.50
*η*	2.75	4.10	0.83	2.00

## Data Availability

The original contributions presented in this study are included in the article. Further inquiries can be directed to the corresponding author.
